# Supplementary material for: The effect of the universal test and treat strategy on the kidney function in adults living with HIV in Zambia: A six-month multicenter cohort study
Source: PLoS One. 2025 May 12;20(5):e0323618. doi: 10.1371/journal.pone.0323618 (PMC12068582; doi:10.1371/journal.pone.0323618)
Supplement: Attachment — Submitted filename: Response to Reviewers.docx [file pone.0323618.s004.docx]

Dr. Lukundo Siame

Luena Road No 21226, Batoka Area,

Livingstone Phone: 0976304745

Email: lukundosiame23@gmail.com Date: 23-09-2024

24^th^ March 2024

To the Editor, PLOS ONE,

Dear Editor,

**Ref: Submission of a revised research article for peer review and publication consideration**

We would like to thank the reviewers for taking the time to make suggestions that have improved our manuscript. We have now revised comments in the manuscript and incorporated all suggestions. We now hope the current manuscript is acceptable for publication. Below are the point-by-point responses to all comments and suggestions.

Editorial comments

1. Please ensure that your manuscript meets PLOS ONE's style requirements, including those for file naming. The PLOS ONE style templates can be found at <https://journals.plos.org/plosone/s/file?id=wjVg/PLOSOne_formatting_sample_main_body.pdf> and <https://journals.plos.org/plosone/s/file?id=ba62/PLOSOne_formatting_sample_title_authors_affiliations.pdf>

**Response:** thank you. done

2. Please include a copy of Table 1, which you refer to in your text on page 5.

**Response:** thank you. done

 3. [Please remove all personal information, ensure that the data shared are in accordance with participant consent, and re-upload a fully anonymized data set.

Note: spreadsheet columns with personal information must be removed and not hidden as all hidden columns will appear in the published file.

Additional guidance on preparing raw data for publication can be found in our Data Policy (https://journals.plos.org/plosone/s/data-availability#loc-human-research-participant-data-and-other-sensitive-data) and in the following article: http://www.bmj.com/content/340/bmj.c181.long.](http://track.editorialmanager.com/CL0/http:%2F%2Fwww.bmj.com%2Fcontent%2F340%2Fbmj.c181.long./1/010f0195abae2fd0-42e372c7-a9ea-44f3-be0f-c842d39ad935-000000/qIafHxk80ZQX3DA4xa8oEwG_C89VwyJlu5WhpjRvoY8=203)

**Response:** thank you. done

4. [Please include captions for your Supporting Information files at the end of your manuscript, and update any in-text citations to match accordingly. Please see our Supporting Information guidelines for more information: http://journals.plos.org/plosone/s/supporting-information.](http://track.editorialmanager.com/CL0/http:%2F%2Fjournals.plos.org%2Fplosone%2Fs%2Fsupporting-information./1/010f0195abae2fd0-42e372c7-a9ea-44f3-be0f-c842d39ad935-000000/wgEfRjEo3xo87ICkClXlJXohiGRDMiDB4eUXfcENc8s=203)

**Response:** thank you. done

**Review Comments to the reviewer.**

Please use the space provided to explain your answers to the questions above. You may also include additional comments for the author, including concerns about dual publication, research ethics, or publication ethics. (Please upload your review as an attachment if it exceeds 20,000 characters)

Reviewer #1: The authors have reported their research findings in the manuscript titled "Universal Test and Treat strategy effect on Kidney Function in Adults living with HIV in Zambia: A Six-Month Multicenter Retrospective Cohort Study". I have the following comments/observations:
1. I suggest the authors should consider rephrasing the title to read "The effect of the universal test and treat strategy on the kidney function in adults living with HIV in Zambia: A six-month multi center cohort study".

**Response:** thank you for the suggestion. We have changed the title.

2. The eGFR calculation formulae used as reported were CKD-EPI in the abstract, and MDRD in the body of the manuscript. The authors should clarify the formula that was actually used.

**Response:** thank you for the suggestion. We have changed the title.

3. In the results section, basic characteristics of study participants (page 5, line 11), the authors mentioned that "a high proportion of participants were taking NNRTI and ABC/3TC regimens. This is similar to what is reported in table 1; however, in table 2, the authors reported 1,173 participants on TDF/XTC, while only **35 participants were on ABC/3TC.** The authors need to resolve these discordant results.

**Response:** thank you for your observations. We have corrected this discordant results in the table.

4. The explanation by the authors to the fact that in the ATT era, participants commenced ART earlier, and did not have renal complications associated with HIV makes scientific sense. However, TDF-based regimens have been reported extensively in literature to be associated with renal impairment in older patients, patients with co-morbidities etc. So it is critical for the authors to clarify whether these cohorts of patients were predominantly on ABC/3TC or TDF/XTC.

**Response:** thank you. we have clarified in the results section and discussion section that most of participants in this study were on TDF/XTC and further discussed the results.

Reviewer #2: Reviewer’s comments on the manuscript titled “Universal Test and Treat strategy effect on Kidney Function in Adults living with HIV in Zambia: A Six-Month Multicenter Retrospective Cohort Study.”

1. Originality of value:
The study is original and will contribute to scientific knowledge and enrich the repository of knowledge on burden of kidney disease among patient living with HIV, its risk factors and how it can be prevented or detected early, particularly in the sub-Saharan African region. The abstract is concisely written while the introduction was appropriately described, and study objective was clearly defined.
2. Suitability and Soundness:
The methodology is appropriate and suitable for the study design.
3. Clarity of presentation:
The manuscript presentation and layout is clear and easy to follow.
4. Areas requiring corrections:
None

**Response:** thank you for appraisal of the manuscript.

Reviewer #3: This article about HIV and kidney disease is timely and pertinent because it highlights the potential for preventing kidney disease. The drawback is the use of eGFR as the sole indicator of kidney impairment. An important hallmark of kidney damage is proteinuria whci was not utilised in this patient. One understands the operational issues with regards to HIV treatment funding and implementation especially with laboratory tests. But if serum creatinine could be done, microalbuminuria/proteinuria could be done.
Older patients would have decline in kidney impairment as per eGFR estimation. Could could the authors provide summary on
of eGFR in the two groups in addition to using it as a cut-off point. Could the trend analysis be done since this since a cohort?

**Response:** thank you. We have now provided the summary of eGFR at baseline and at the endline of the study. Unfortunately, we are unable to do the trend analysis due to the nature in which the study was conducted. We only collected data at two points at baseline of the study and at 6 months. In addition, our result in tables two and three point to the fact that older individuals were at risk of kidney impairment.

The explanation for kidney impairment among urban dwellers is far-fetched, especially when this is not based on the data from the study. There is no evidence that these patients had hypertension or diabetes. It may just reflect the disproportionately large number of urban dwellers in the study.

**Response:** thank you. We have revised the explanation of as observed, indeed this was might have been a reflection due to disproportionately large numbers of urban dwellers in this study.

We have revised the manuscript and addressed all concerns raised by the reviewers. We want to thank you all again for the tremendous work and time that you committed in editing our work. Our manuscript is much improved, and we are very grateful.

Yours sincerely,

Dr. Lukundo Siame, Bsc., MBcHB.

Junior Residence Medical Livingstone University Teaching Hospital
